# Application and Validation of a transRADial Access Score (RAD-Access) in Patient Selection for Safe Radial Access in Liver Cancer Intra-Arterial Procedures

**DOI:** 10.3390/cancers17091385

**Published:** 2025-04-22

**Authors:** Roberto Iezzi, Alessandro Posa, Andrea Contegiacomo, Alessandro Maresca, Elena Rodolfino, Biagio Merlino, Tiago Bilhim, Marcelo Guimaraes

**Affiliations:** 1 Department of Diagnostic Imaging, Oncologic Radiotherapy, and Hematology, Fondazione Policlinico Universitario A. Gemelli–IRCCS, Largo A. Gemelli 8, 00168 Rome, Italy; roberto.iezzi@policlinicogemelli.it (R.I.); andrea.contegiacomo@policlinicogemelli.it (A.C.); alessandro.maresca@guest.policlinicogemelli.it (A.M.); elena.rodolfino@policlinicogemelli.it (E.R.); biagio.merlino@policlinicogemelli.it (B.M.); 2 Facoltà di Medicina e Chirurgia, Università Cattolica del Sacro Cuore–Sede di Roma, Largo F. Vito 1, 00168 Rome, Italy; 3 Interventional Radiology Unit, Curry Cabral Hospital, Unidade Local de Saùde Sao José, Centro Clinico Academico de Lisboa, R. Beneficiencia 8, 1069-166 Lisbon, Portugal; tiagobilhim@hotmail.com; 4 Division of Vascular and Interventional Radiology, Department of Radiology, Medical University of South Carolina, Charleston, SC 29425, USA; interventionalmc@gmail.com

**Keywords:** transradial, chemoembolization, radioembolization, prediction, score

## Abstract

**Objectives:** To develop and internally validate a pre-treatment score for the safe selection of the best candidates for the transradial approach when performing liver cancer intra-arterial procedures. **Methods:** One hundred and twenty-two patients undergoing hepatic endovascular treatments via radial access between January and December 2022 were retrospectively selected to develop a prediction model. Pre-procedural imaging data were analyzed, and variables were selected to develop the RAD-access score. Intra-procedural data were analyzed to assess effective procedural complexity (ePC). The relationship between ePC and pre-procedural variables was statistically tested, and cutoff points were defined. **Results:** A final score (RAD-access) was created and prospectively validated on 139 patients enrolled between June and September 2023. Aortic arch diameter and angulation, left subclavian artery angulation, suprarenal abdominal aorta diameter, celiac trunk take-off angle, and radial artery diameter were the significant variables used to build the score. In the validation cohort, based on the pre-treatment RAD-access score, 69 patients underwent a transradial approach, with a significantly lower ePC rate obtained (78.2% easy, 20.3% intermediate, 1.5% complex). No major adverse events occurred. **Conclusions:** Pre-treatment RAD-access score provides a good prediction for the procedural complexity of the transradial approach in patients undergoing liver cancer intra-arterial treatments, identifying the best candidates for an easy and safe transradial procedure.

## Simple Summary

The aim of this study is to develop a useful and easy to apply score to choose the best candidates for transradial access for liver cancer intra-arterial procedures, by predicting the procedural complexity based on anatomical measurements on pre-procedural CT examination.

## 1. Introduction

Intra-arterial procedures have been acknowledged to play an important role in the treatment of both primary and secondary liver neoplastic lesions, becoming an inextricable aspect of every therapeutic algorithm [1,2,3,4]. Regardless of the type of procedure performed and the superselective or lobar approach, most procedures are still performed through a common femoral artery access.

Transradial access, on the other hand, incurs less patient discomfort, without bed restrictions, with a short learning curve and similar procedural times and patient radiation doses when compared to the standard trans-femoral access [5,6,7]. This approach is widely adopted in cardiology, and is also being applied more and more in current interventional radiology and interventional oncology practice [5,6,7]. However, in this scenario, the choice of a femoral or radial approach is currently based only on the operator’s experience or preference, as there is a lack of objective pre-procedural evaluation criteria that could be used to identify the patients to select for liver cancer intra-arterial procedures in which a transradial approach could be easily performed.

Based on this background, our study wants to address the lack of knowledge in current interventional oncology practice, aiming to develop and internally validate a pre-treatment score (RAD-access), based on anatomical measurements from computed tomography (CT) imaging and radial artery ultrasound, for the safe selection of the best candidates for the transradial approach when performing liver cancer intra-arterial procedures.

## 2. Materials and Methods

### 2.1. Study Design and Patients

This study was conducted in accordance with the declaration of Helsinki. All patients signed an informed consent for the procedure prior to the examination.

Two different cohorts of patients were considered: one cohort—retrospectively enrolled from January to December 2022—was used to develop the prediction model (the development cohort) and the other—prospectively enrolled from June to September 2023—was used to validate and assess the abilities of the model (the validation cohort).

### 2.2. The Development Cohort

All consecutive patients with primary or secondary hepatic tumors, who underwent intra-arterial hepatic locoregional treatments (chemoembolization, bland embolization, or radioembolization) performed by a single experienced interventional radiologist, with more than 15 years of experience using a transradial approach, were retrospectively enrolled between January and December 2022. In the retrospective development cohort, transradial approach was selected based on operator’s preference at the time of the procedure, usually excluding patients (a) with absence of pulse or abnormal Allen or Barbeau test (curve D) in left radial artery, (b) with presence of dialysis fistula or impending dialysis dependence in left arm, or (c) who refused a transradial approach. In the last four years, in clinical practice, based on experience and dedicated devices available, radial artery caliber as well as patients’ characteristics (height, BMI) are not considered absolute exclusion criteria.

The patients were identified by searching both the Radiology Information System (RIS) and Picture Archiving and Communication System (PACS). The research was performed by two interventional radiologists with more than 3 years of experience and the keywords “embolization” and “hepatic tumor” were used. Patients’ clinical and procedural data and characteristics were subsequently retrieved from electronic medical records and collected by the same two radiologists. Patients with missing intra/post-procedural parameters or missing arterial phase thoraco-abdomino-pelvic CT images obtained within 3 months before the locoregional treatment were excluded from the present study.

Based on pre-procedural parameters, a scoring system for selecting the best candidates for the transradial approach in which the liver cancer intra-arterial procedures could be easily performed was developed.

#### 2.2.1. Patient Demographics and Procedural Parameters

The following clinical parameters were retrieved and collected: patient characteristics (age, sex, body mass index [BMI], and height). The following procedural parameters on transradial access were also evaluated: previous radial artery puncture and radial artery diameter (RD) (measured at the pre-procedural ultrasound examination). Data are shown in Table 1.

#### 2.2.2. Pre-Procedural CT Imaging Parameters

Pre-procedural CT examinations were retrospectively analyzed independently by two radiologists, each one with more than five years of experience in thoraco-abdominal radiology. Readers were blinded to medical history, clinical parameters, and procedural details. The following data were collected for all the CT examinations performed prior to the procedure: aortic arch diameter (AAD, measured at the level of origin of subclavian artery) and angulation (AAA) according to Madhwal and colleagues [8]; left subclavian artery angulation (LSA); descending thoracic aorta diameter evaluated in the proximal tract; angulation of descending thoracic aorta (according to the classification by Belvroy and colleagues [9]; suprarenal abdominal aorta diameter (SAD) measured at the level of origin of celiac trunk; take-off angle of celiac trunk (CTA); anatomical variants of the hepatic artery [10,11]; and previous aortic and periaortic vessel interventions, if any. The data sets of each patient were loaded into a dedicated three-dimensional workstation (Advantage Workstation VolumeShare 4; GE Healthcare, Milwaukee, WI, USA). For length measurements, reformatted cross-sectional images longitudinal to the long axis of the vessel were automatically obtained to create “modified coronal” planes. Vessel segmentation was automatically performed. All measurements of diameters, lengths and angles were manually completed using graphical measurement tools in the post-processing application software. To minimize variability, the two experienced radiologists performed all measurements twice, and the final mean values were considered.

Liver lesions’ location (lobe and segment) and treatment strategy (lobar or selective embolization) were also registered. Data are shown in Table 1.

#### 2.2.3. Effective Procedural Complexity of Radial Access

Effective procedural complexity was classified using a three-point scale of easy, intermediate, and complex. Procedural complexity (ePC) was defined based on evaluation of angiographic and procedural variables obtained for the entire study population (development cohort) (time of puncture—from anesthetic administration at the site of vascular access until placement of the arterial sheath; number of punctures; fluoroscopy time; total time of examination—from arterial sheath placement to its removal) performed by two experienced interventional radiologists with more than 15 years’ experience. To maximize the correct evaluation of access-related complexity, to each variable was assigned a score of 0 (if less than the mean value of the variable obtained for the entire population), 1 (if equal to the mean value), whereas, if higher than the mean value, 3 points for time of puncture and number of punctures—that are directly influenced by transradial access—and 2 points for fluoroscopy time and total time of examination—that are not only influenced by transradial access—with a maximum of 10 points available (Table 2).

An additional 2 points were assigned in case of procedural mild adverse event being registered, or where the procedure was considered at all complex in the case of a moderate, severe, life-threatening or disabling access-related adverse event being registered; procedural adverse events were classified according to Society of Interventional Radiology Specialty–Specific System [12]. The procedure was also considered complex in the occurrence of a conversion from radial access to femoral access.

**Figure 1:**
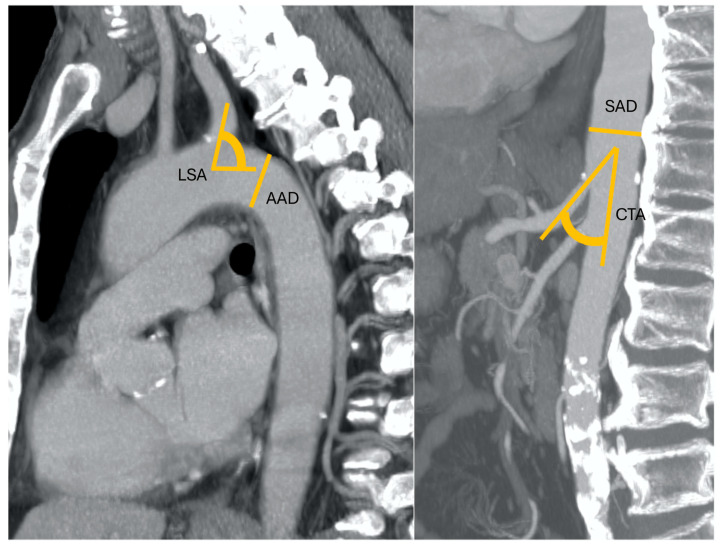
A 52-year-old patient with nodular HCC in the left liver lobe with a radial diameter (RD) of 3 mm (0 points), left subclavian artery angle (LSA) of 85° (0 points), an aortic arch angulation (AAA) type I (0 points) with a diameter (AAD) of 32 mm (1 point), suprarenal aorta diameter (SAD) of 23 mm (0 points), and celiac trunk angle (CTA) of 45° (0 points). Final RAD-access score: 0, transradial access preferred for values between 0 and 2.

**Figure 2:**
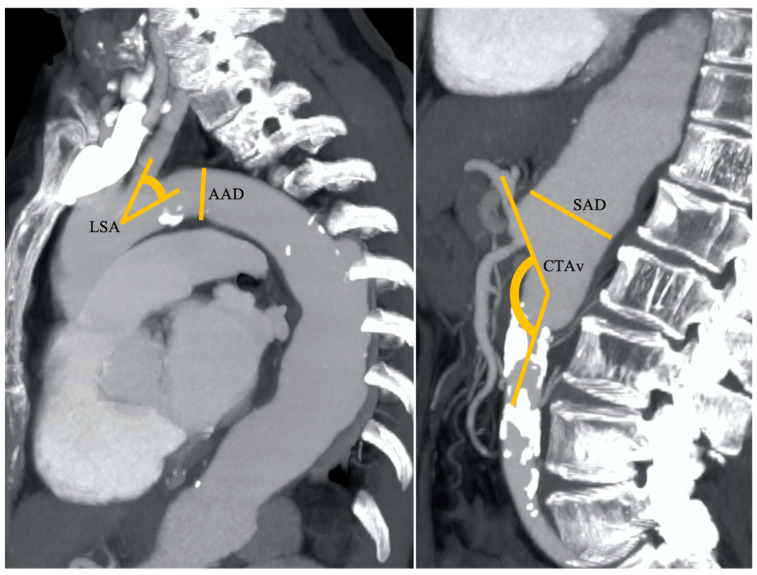
An 83-year-old patient with nodular HCC in the right liver lobe with a radial diameter (RD) of 2 mm (not shown) (0 points), left subclavian artery angle (LSA) of 45° (1 point), an aortic arch angulation (AAA) type III (1 point) with a diameter (AAD) of 32 mm (1 point), suprarenal aorta diameter (SAD) of 40 mm (1 point), and celiac trunk angle (CTA) of 130° (1 point). Final RAD-access score: 5, transfemoral access preferred for values between 3 and 6.

The procedure was considered “easy” if a score of less than 3 points was obtained plus the absence of procedural adverse events (Figure 1); the procedure was considered “intermediate” in the case of a score between 3 and 5 points, including mild adverse event analysis; the procedure was considered “complex” if a score higher than 5 was obtained and/or in the case of a significant (moderate, severe, life-threatening or disabling) access-related adverse event (Figure 2).

It is mandatory to highlight that the expected rate of stroke and TIA is generally low enough that the current study is underpowered to study them.

### 2.3. The Validation Cohort

All consecutive patients with primary or secondary hepatic tumors referred to the Institution to be treated with liver cancer intra-arterial treatment between June and September 2023 were prospectively evaluated. The same experienced interventional radiologist, who was involved in the development cohort, selected the procedural approach based on the pre-treatment RAD-access score. In detail, a trans-radial approach was selected in patients with a RAD-access score up to 2, whereas the other patients (RAD-access score more than 2) were treated with a transfemoral approach.

For the included patients, who underwent transradial treatments, the angiographic and procedural variables (time of puncture—from anesthetic administration at the site of vascular access until placement of the arterial sheath; number of punctures; fluoroscopy time; total time of examination—from arterial sheath placement to its removal) as well as procedural adverse events or conversion from radial to femoral access were registered for evaluating the effective procedural complications.

### 2.4. Radial Access Technique

The left radial artery was usually preferred, punctured using ultrasound guidance, with local anesthesia. Dedicated devices were always used, as 5Fr Glidesheath Slender (Terumo Corp, Tokyo, Japan) vascular introducer sheath, 110 cm Optitorque Multipurpose (Terumo Corp) (4 cm length tip), and 2.4/2.7-F microcatheters (Progreat; Terumo Corp). To prevent vasospasm and reduce the risk of clot formation, a mix of 2.5 mg of verapamil, and 1000 IU of heparin was injected intra-arterially through the vascular sheath.

### 2.5. Statistical Analysis

Data were collected in an Excel database (Excel 356, Microsoft Corporation, Redmond, WA, USA), and statistical analysis was performed with SPSS software version 23. Continuous variables were reported as mean with standard deviation, categorical variables as frequencies with percentages. Moreover, statistical analysis was carried out using correlation coefficient calculations, linear regression as well Spearman’s Rank correlation coefficients and ANOVA for each continuous variable to explore data, also using Kruskal–Wallis’ test, when Levene’s Test for Equality of Variances was significant.

The relationships between procedural complexity and categorical variables were tested with χ^2^ test using *p*-values under or equal to 0.05 as statistically significant. Binary logistic regression analysis including all significant variables was performed. The best cutoff points were defined using ROC curves through calculations of Youden Indexes [13]. A final score, named the transRADial access score (RAD-access), was created. For each variable, values of 0 or 1 were attributed based on the cut-off thresholds derived by Youden’s ROC curves. RAD-access scores were calculated by the sum of all values.

For internal validation, the developed score was applied to the validation cohort. The relationship between predicted and observed procedural complexity was indicated for calibration.

To calculate sensitivity and specificity of the RAD-access score in association with procedural complexity, a 2 × 2 table was constructed based on procedural complexity of 1 or 2 versus 3, and RAD-access score cutoff value of 0–2 versus 3–6.

## 3. Results

### 3.1. The Development Cohort

#### 3.1.1. Study Population

During the study period, a total of 587 intra-arterial locoregional treatments for primary or secondary liver tumors were performed. One hundred eighty-two procedures were performed using a transradial approach (31%), based on the operator’s decision. Technical success was obtained in all patients with no switch from radial access to femoral access during any procedure (crossover rate 0%).

A total of 60 patients were excluded from the present study; in detail, clinical and/or procedural parameters were missing in 31 patients whereas CT images were not available (inadequate or uncompleted) for the last 29 patients.

A total of 122 patients were included in the retrospective cohort and had the RAD-access score evaluated.

#### 3.1.2. Effective Procedural Complexity

Table 2 demonstrates the mean angiographic and procedural variable results for the development cohort. Regarding post-procedural adverse events, there were no major vascular or neurological adverse events (0%).

Four mild adverse events (4/122, 3.3%) were observed (one puncture site hematoma and three ecchymoses), all self-limited without any further intervention (percutaneous or surgical) or any clinical sequelae. No signs of hand ischemia or radial pulse absence were observed after the procedure and at the clinical and US examination performed at 1-month follow-up. Based on procedural evaluation, for the development cohort, the effective procedural complexity (ePC) was registered as easy in 25 procedures (25/122, 20.5%), intermediate in 69 (69/122, 56.6%), and complex in 28 procedures (28/122, 22.9%).

#### 3.1.3. Clinical, Procedural and Pre-Procedural CT Imaging Parameters

The results regarding clinical, procedural and pre-procedural CT parameters are shown in Table 1.

No substantial differences were observed in sex, age, or type and location of procedure. In 51 patients (41.8%) the radial access had already been previously used, without differences between the three procedural complexity groups. The radial diameter was significantly smaller in the complex ePC group (*p* < 0.001). Among the pre-procedural CT imaging parameters, the aortic arch diameter and angulation (type III), left subclavian artery angulation, suprarenal abdominal aortic diameter and take-off angle of the celiac trunk were correlated to the effective procedural complexity values, with statistical significance when comparing the values of easy and intermediate ePC and complex ePC (*p* < 0.001, Table 1). No significant differences were observed for the other parameters considered.

#### 3.1.4. RAD-Access Score Definition

All parameters in the development cohort correlating with the ePC (radial artery diameter, RD; AAD, AAA; LSA; SAD; CTA) were considered to produce the RAD-access score.

**Figure 3:**
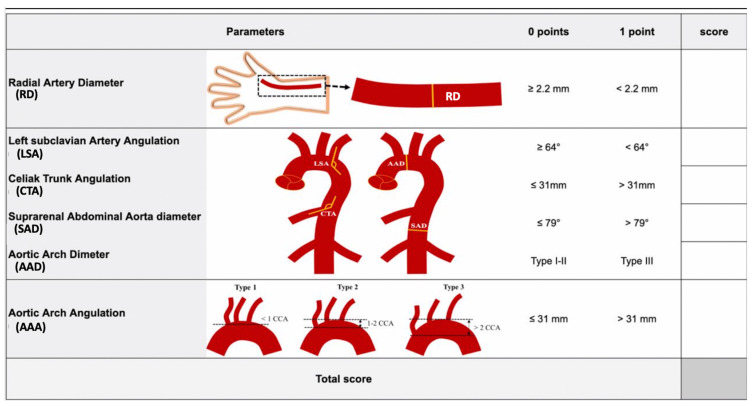
RAD-access score evaluation. CCA: common carotid artery diameter.

For all the parameters except AAA, the ROC curves were calculated estimating the Youden J index and the associated criterion for discriminating against easy/intermediate/complex procedural complexity. The cut-off corresponding to the maximum value of Youden’s index for the ROC curve was used to calculate the value for each variable (0 or 1) to be included in the sum producing the final RAD-access score. Finally, for AAA, a score of 1 was given only in the case of type III angulation. Therefore, a RAD-access score ranging from 0 to 6 was calculated according to the formula: RAD-access score = RD + SAD + CTA + LSA + AAD + AAA, for each of the added possible values being 0 or 1, and compared with PC values (Table 3, Figure 3).

Considering a RAD-access score threshold value >2, out of the 69 intermediate ePC cases, only 1 case was overestimated, whereas 5 (19.8%) were underestimated, with an overall sensitivity of 82.1%, an overall specificity of 98.9%, and an overall accuracy of 95.1% (*p* < 0.001).

### 3.2. The Validation Cohort

A total of 153 patients with indication for hepatic intra-arterial procedures, potential candidates for either approach (transradial or transfemoral), were enrolled during the study period (Table 4).

The RAD-access score was obtained for 139 patients. It was not calculated in 14 patients (9.1%) due to missing data (no previous thorax CT examinations available). US evaluation of radial diameter was performed before treatment. The mean time for RAD-access score evaluation (that included US evaluation of radial artery and CT data) was 13.5 ± 6.4 min.

A RAD-access score of 0 was obtained in 35 patients (25.2%), a score of 1 in 19 patients (13.7%), a score of 2 in 15 patients (10.8%), a score of 3 in 21 patients (15.1%), a score of 4 in 19 patients (13.7%), a score of 5 in 19 patients (13.7%), and a score of 6 in 11 patients (7.9%).

Based on the RAD-access score, a total of 69 patients (49.6%) with a score up to 2 were prospectively identified to be treated with a trans-radial hepatic intra-arterial procedure, with technical success obtained in all patients, without conversion from radial to femoral access (cross-over rate: 0%). No major vascular or neurological adverse events (0%) were registered, with only two minor adverse events (2.9%) represented by two puncture site ecchymosis, both self-limiting, without any clinical sequelae. No signs of hand ischemia or radial pulse absence were observed after the procedure or at the one-month clinical and US evaluation.

The effective procedural complexity for transradial procedures calculated for the 69 patients included in the validation cohort was considered as easy in 54 cases (54/69, 78.2%), intermediate in 14 cases (14/69, 20.3%), and complex in 1 case (1/69, 1.5%). Angiographic and procedural results obtained in these patients are in Table 4.

The only complex transradial procedure—based on a longer fluoroscopy time (510 s) and longer total time of examination (66 min), but without any procedural adverse events —was registered in a patient with a pre-procedural RAD-access score of 2, with large suprarenal abdominal aorta (SAD = 46 mm, 1 point) and celiac trunk take-off angle of 124° (1 point). The predicted probability of complexity was well calibrated to the observed intraprocedural evaluation. The RAD-access score calculated the net benefit in performing an easy trans-radial approach in 78.2%, with a complex procedure registered in only 1.5%.

The last 70 patients with a RAD-access score higher than 2 underwent an intra-arterial hepatic procedure using a transfemoral approach.

### 3.3. Development Versus Validation Cohort

Comparison between the two experimental cohorts is reported in Table 5.

In the validation group, a significantly larger radial artery, smaller aortic arch and suprarenal aortic diameters, higher left subclavian angle, lower celiac trunk angle and higher number of type III aortic arch angulations were registered. No significant differences in terms of safety were found. Lower fluoroscopy and total examination time were registered as well as a shorter time and lower number of radial punctures were obtained in the validation group. When compared to the development cohort, considering the ePC, a significantly higher rate of easy procedures (78.2% vs. 20.5%) and a lower rate of complex procedures (22.9% vs. 1.5%) were registered in the validation cohort.

## 4. Discussion

The transradial approach has been adopted as the first-line approach for most coronary interventions; however, it remains underused by interventional radiologists.

Many authors demonstrated that the transradial approach is safe, effective, and preferred by patients, due to less post-procedural discomfort with faster patient ambulation/discharge, when compared to TFA, so that it could be considered as the primary arterial access [5,6,14,15,16,17,18,19,20,21,22].

However, being all about patient comfort and safety, it would not be correct to push radial too hard (switching all the procedures from femoral to radial) if it may not be the best option for the patient (if transradial approach would be too complex).

To the best of our knowledge, there are no validated pre-procedural criteria that could help operators in selecting the best candidates for the radial approach for intra-arterial hepatic procedures regardless of their experience or preference. Therefore, the development of a scoring system for predicting a safe and easy radial access in liver cancer endovascular procedures could be valuable.

It is mandatory to underline that not a single parameter is able to define the expected procedural complexity and the selection of access.

In the development cohort, through regression coefficients analysis, a RAD-access score was developed with the goal of predicting radial access procedural complexity and tailoring a patient-centered decision during the vascular access selection.

In the multivariable regression analysis, six variables were significant predictors of procedural complexity, represented by RD value and CT variables such as LSA, AAA and AAD, SAD, CTA.

No differences were found when considering the presence or absence of previous radial punctures, or the procedure type (e.g., lesion location, lobar/selective treatment). Those variables could have had no impact due to the use of vasodilators and heparin (which can reduce the risk of clot formation), the availability of long diagnostic catheters designed for radial access and of long microcatheters facilitating selective and superselective hepatic artery catheterizations. The availability of long diagnostic catheters and microcatheters also helps in overcoming issues related to patient height, which must not represent a limitation in performing radial access.

The first variable to be evaluated was RD. Although not an inclusion/exclusion criterion in experienced centers or for well-trained operators, the literature demonstrates that the smaller the size of the radial artery, the harder it may be to obtain vascular access (more complex) [23]. This can be particularly true in female patients, due to the overall small size when compared to male patients; luckily, the use of low-profile radial introducer sheaths and catheters has helped to overcome this limitation. Moreover, RD should always be measured, as it is not the only parameter which determines the procedural complexity: if the RD is less than 2.2 mm but the other parameters are in favor of a transradial approach, the operator should consider it as an option (given that Barbeau’s and Allen’s tests are good), as a single parameter is not able to define the expected procedural complexity and lead access selection [5,7].

The LSA and the CTA significantly impact the procedural complexity, because their orientation could allow a fast catheterization of the descending thoracic aorta from the left subclavian artery and a selective catheterization of the celiac trunk due to its downward orientation from the abdominal aorta. These two angles can maximize coaxial cannulation, improving support, making the transradial procedure easier and faster. AAA and AAD can also determine the difficulty in catheterization influencing catheter performance. Anatomical geometry and the caliber of the aortic arch constrain catheter motion, potentially influencing transradial complexity. A catheter approaching the descending thoracic aorta encounters two types of turns: moderate angles branching from the subclavian artery and angle at the aortic arch. The tip angulation of the catheter, in conjunction with the turn on the wire, partially affects the ability to catheterize the descending aorta, which is mainly influenced by aortic arch diameter. SAD—the last parameter—is indirectly correlated with an easy celiac trunk cannulation, as the larger calibers lead to a more complex procedure.

In the internal validation cohort, the RAD-access score was able to guide the operators in selecting patients with a high probability of obtaining a safe and easy trans-radial procedure, as demonstrated by the higher rate of easy procedures performed (78.2%) in the validation cohort (versus 20.5% in the development cohort), with only one procedure considered complex (1.5%), with no procedural adverse events registered. Furthermore, the score was easy and fast to execute, with a mean time of less than 15 min (13.5 ± 6.4 min), including the pre-procedural ultrasound radial evaluation.

From a methodological standpoint, as score generalizability is an important concern, the two main drawbacks of this study are that our RAD-access score was generated by using retrospective single-institution data based on a single operator and for a limited number of liver cancer intra-arterial procedures only, with no external validation; these two aspects could generate potential selection and validation biases. Furthermore, another potential limitation of the study is that in the validation group, a transradial approach was performed only in the case of a RAD-access score up to two, to confirm the prediction of an easy procedure. Therefore, a large prospective multicenter external validation is needed in order to confirm our data, involving multiple operators, different centers, and different procedures, excluding any inclusion or procedural bias. The RAD-access score could also be used by physicians as a steppingstone to identify further radial access parameters and create radial access scores for various procedures, broadening its application beyond hepatic procedures.

Another limitation of this study is that it only addresses the transradial approach performed through the left radial artery and only includes the LSA among the variables for the calculation of the score; therefore, it has limited applicability in the case of right radial access as it does not consider the anatomy and angulation of the brachiocephalic trunk. However, the great majority of transradial procedures for liver cancer treatment are performed through a left radial artery; nonetheless, this issue can be addressed in further studies.

A potential drawback of the proposed score would also be the potential lack of a thoracic CT scan of patients with liver cancer, diminishing the possibility of calculating the score. However, all patients with liver metastases usually undergo multidetector-row CT covering the entire abdomen and chest for staging and follow-up and in recent years, a complete work-up of HCC patients also includes dynamic contrast-enhanced CT for staging, excluding extrahepatic disease. This concept was also confirmed in the validation cohort in which the RAD-access calculation was not available in only 9.1% of patients.

## 5. Conclusions

The RAD-access calculation model produces a complexity score to improve vascular access selection in the intra-arterial procedures of patients with primary and secondary liver cancer. A transradial approach seems preferable for a RAD-access score up to two, indicating a low procedural complexity and potentially better procedural outcomes. This model was demonstrated to be helpful for selecting appropriate candidates for intra-arterial hepatic procedures performed via the transradial approach even if it needs external validation. The RAD-access score could be useful for experienced operators and crucial for operators looking to expand their skills in the transradial approach avoiding complex cases.

## Figures and Tables

**Table 1:** Clinical and procedural parameters.

Code	Study Group	PC 1	PC 2	PC 3	ANOVA/Chi Square (*p*)	Levene	Kruskal–Wallis (*p*)	Rho CC	Spearman Rank (*p*)
SEX	122 patients: 82 M, 40 F (68.7 ± 8.3 yrs)	17 M, 8 F (67.5 ± 6.5)	41 M, 28 F (68.3 ± 9.2)	24 M, 4 F (70.6 ± 7.4)	0.049				
PROC	primary/secondary liver tumor embolization (58 lobar, 48 segmental, 16 subsegmental)	14/6/5	34/29/6	10/13/5	0.228				
LS	61 right, 61 left (50.0%)	13/12	34/35	14/14	0.973				
RPP	51/122 (41.8%)	11/25	28/69	12/28	0.949				
RD	2.6 ± 0.7 mm	2.8 ± 0.7	2.7 ± 0.5	2.2 ± 0.8	<0.01	0.081	<0.001	−0.284	0.0016
AAA	Type I 52, Type II 51, Type III 19	12/13/0	36/28/5	4/10/14	<0.0001				
AAD	29.5 ± 3.3 mm	27.9 ± 2.2	29.0 ± 2.5	32.3 ± 4.0	<0.001	0.001	<0.001	0.402	<0.0001
LSA	69.1 ± 7.0 mm	72.1 ± 7.3	70.8 ± 5.4	62.4 ± 6.3	<0.001	0.154	<0.0001	−0.480	<0.0001
TAP	9/122 (7.4%)	22/3	63/6	28/0	0.203				
TAD	31.8 ± 4.2 mm	31.0 ± 3.4	31.1 ± 3.8	34.1 ± 4.8	0.003	0.061	0.011	0.204	0.0241
TAA	low 53, intermediate 48, high 31	9/10/6	34/28/7	10/10/8	0.219				
SAD	30.9 ± 3.7 mm	29.0 ± 1.6	29.8 ± 2.3	35.3 ± 4.5	<0.001	<0.001	<0.0001	0.511	<0.0001
CTA	67.9 ± 22.7 mm	58.9 ± 16.5	62.1 ± 16.6	90.1 ± 26.3	<0.001	0.003	<0.001	0.420	<0.001
HA	12/122 (9.8%): 5 LHA from LGA, 7 RHA from SMA	5	6	1					

Table legend: PC = procedural complexity; PROC = procedure type; LS = lesion Side; RPP = radial previous puncture; RD = radial artery diameter; AAD = aortic arch diameter; AAA = aortic arch angulation type; LSA = left subclavian artery angle; TAP = thoracic aorta previous treatment; TAD = thoracic aorta diameter; TAA = thoracic aorta angle; SAD = suprarenal aorta diameter; CTA = celiac trunk angle; HA = anatomical variants of hepatic artery; LHA = left hepatic artery; LGA = left gastric artery; RHA = right hepatic artery; SMA = superior mesenteric artery.

**Table 2:** Mean angiographic and procedural results used to calculate effective procedural complexity (ePC).

	Mean Value (1 Point)	Lower (0 Point)	Higher Value (* 2 Points-§ 3 Points)
Time of puncture (min)	4.23 ± 1.23	<4	>5 §
Number of punctures	1.3 ± 0.64	1	>2 §
Fluoroscopy time (s)	395 ± 74.34	<300	>460 *
Total time of examination (min)	48.43 ± 16.74	<35	>60 *

**Table 3:** Sensitivity, specificity and accuracy of each variable included in TASS calculation based on defined cut-off values.

	Parameters		RD	LSA	SAD	CTA	AAA	AAD	
	**Cut-off values**		<2.2 mm	<64 mm	>31 mm	>79 mm	type III	>31 mm	
	**PC < = 2**		84	96	88	95	103	95	
	**PC > 2**		38	26	34	27	19	27	
	TP		19	20	21	17	17	14	
	TN		75	88	81	84	84	89	
	FP		19	6	13	10	10	5	
	FN		9	8	7	11	11	14	
	sens		67.9%	71.4%	75.0%	60.7%	50.0%	60.7%	
	spec		79.8%	93.6%	86.2%	89.4%	94.7%	89.4%	
	acc		77.0%	88.5%	83.6%	82.8%	84.4%	82.8%	
			122	122	122	122	122	122	
	**TASS—Development Cohort**								
		**0**	**1**	**2**	**3**	**4**	**5**	**6**	
**PC**	**1**	11	13	1	0	0	0	0	25
	**2**	33	25	10	1	0	0	0	69
	**3**	2	2	1	5	6	7	5	28
		46	40	12	6	6	7	5	**122**
		*p* < 0.0001							
				sens	**82.1%**				
				spec	**98.9%**				
				acc	**95.1%**				
	**TASS—Validation Cohort**								
		**0**	**1**	**2**	**3**	**4**	**5**	**6**	
**PC**	**1**	34	14	6	0	0	0	0	
	**2**	1	5	8	0	0	0	0	
	**3**	0	0	1	0	0	0	0	
		35	19	15	0	0	0	0	

For each variable, values 0 or 1 were attributed on the basis of the cut-off thresholds derived by Youden’s ROC curves, TASS calculated by the sum of all values. For sensitivity, specificity and accuracy, a cut-off between 1, 2 and 3 groups was considered for PC, based on statistics showing significative differences between those groups. Table legend: RD = radial artery diameter; LSA = left subclavian artery angle; SAD = suprarenal aorta diameter; CTA = celiac trunk angle; AAA = aortic arch angulation type; AAD = aortic arch diameter; PC = procedural complexity; TP = true positives; TN = true negatives; FP = false positives; FN = false negatives. Sens = sensitivity; Spec = specificity; Acc = accuracy.

**Table 4:** Clinical and procedural parameters for validation cohort.

	Non TRA (70)	TRA (69)	*p*
Sex (male)	50 (71.4%)	47 (68.1%)	0.093
Age (years)	72.3 ± 5.2	65.9 ± 5.6	0.062
Procedure type (L/S/SS)	15/31/24	19/28/22	0.098
Disease			0.128
HCC	52 (74.3%)	43 (62.3%)	
mCRC	13 (18.6%)	15 (21.7%)	
Others	5 (7.14%)	11 (15.9%)	
Treated lesion side (right)	32 (45.7%)	42 (60.8%)	0.062
RPP	23 (32.8%)	24 (34.8%)	0.074
RD (mm)	1.9 ± 0.8	2.8 ± 0.3	**0.018**
AAA (Type I-II/III)	51/19	67/2	**0.011**
AAD (mm)	38.5 ± 8.6	27.3 ± 4.1	**0.002**
LSA	59.5 ± 7.4	76.3 ± 5.4	**0.034**
SAD (mm)	37.2 ± 6.4	27.1 ± 6.2	**0.002**
CTA	84.6 ± 95.2	63.6 ± 12.8	**0.001**
Time of puncture (min)	-	3.23 ± 0.84	N/A
Number of punctures	-	1.1 ± 0.34	N/A
Fluoroscopy time (sec)	-	335 ± 59.24	N/A
Total time of examinations (min)	-	38.23 ± 9.64	N/A

Table legend: TRA = transradial approach; L = lobar; S = segmental; SS = subsegmental; HCC = hepatocellular carcinoma; mCRC = metastatic colorectal cancer; RPP = radial previous puncture; RD = radial diameter; AAA = aortic arch angulation type; AAD = aortic arch diameter; LSA = left subclavian angle; SAD = suprarenal aortic diameter; CTA = celiac trunk angle.

**Table 5:** Comparison between development and validation cohort.

	Development Cohort (122 pts)	Validation Cohort (69 pts)	*p*
Sex (male)	82 (67.2%)	47 (68.1%)	0.751
Age (ys)	68.7 ± 8.3	65.9 ± 5.6	0.103
Procedure yype (L/S/SS)	58/48/16	19/28/22	0.042
RPP	51 (41.8%)	24 (34.8%)	0.039
RD (mm)	2.6 ± 0.7	2.8 ± 0.3	0.081
AAA (Type I-II/III)	103/19	67/2	0.001
AAD (mm)	29.5 ± 3.3	27.3 ± 4.1	0.073
LSA	69.1 ± 7	76.3 ± 5.4	0.038
SAD (mm)	30.9 ± 3.7	27.1 ± 6.2	0.028
CTA	67.9 ± 22.7	63.6 ± 12.8	0.018
Time of puncture (min)	4.23 ± 1.23	3.23 ± 0.84	0.002
Number of punctures	1.3 ± 0.64	1.1 ± 0.34	0.043
Fluoroscopy time (sec)	395 ± 73.34	335 ± 59.24	0.023
Total time of examinations (min)	48.43 ± 16.74	38.23 ± 9.64	0.001
Technical success	100%	100%	-
Cross-over/major complications	0%	0%	-
Minor complications	3.28% (4/122)	2.9% (2/69)	0.139
Procedural complexity			
1—Easy	20.5% (25)	78.2% (54)	0.001
2—Intermediate	56.55% (69)	20.3% (14)	0.002
3—Complex	22.95% (28)	1.5% (1)	0.001
RAD-access score			
0	37.7% (46)	49.2% (34)	0.041
1	32.8% (40)	31.9% (22)	0.356
2	9.8% (12)	18.8% (13)	0.024
3	4.9% (6)	-	
4	4.9% (6)	-	
5	5.7% (7)	-	
6	4.1% (5)	-	

Table legend: L = lobar; S = segmental; SS = subsegmental; RPP = radial previous puncture; RD = radial diameter; AAA = aortic arch angulation type; AAD = aortic arch diameter; LSA = left subclavian angle; SAD = suprarenal aortic diameter; CTA = celiac trunk angle.

## Data Availability

Data will be made available on request.
